# Electroconvulsive Therapy in Functional Hallucination: Scope and Challenges

**DOI:** 10.1155/2017/9421973

**Published:** 2017-10-17

**Authors:** Sulochana Joshi, Rabi Shakya

**Affiliations:** Department of Psychiatry, Patan Academy of Health Sciences, Lalitpur, Nepal

## Abstract

Functional hallucinations are hallucinations triggered by a stimulus in the same modality and cooccur with it. They are rare in occurrence; however, their rarity has no significance as psychopathology till date. Also, very little is known about the treatment of such hallucinations. Electroconvulsive therapy (ECT) has been tested for several psychiatric illnesses and has a few relative contraindications; however, it has not previously been used in treating functional hallucinations. We report on a female patient with paranoid schizophrenia who experienced functional hallucinations continuously despite the use of adequate risperidone, which controlled other symptoms. She was treated with ECT which resolved the functional hallucinations. The case highlights the need to ponder on the significance of the phenomenon as well as treatment of this psychopathology by ECT. It also underscores ECT as a treatment option for this kind of hallucination.

## 1. Introduction

Functional hallucinations are a form of perceptual disorder in which hallucinations are triggered by a stimulus in the same modality and cooccur with it [1]. Their exact significance is unknown, but they have been reported in schizophrenia and other psychotic disorders. Compared with hallucinations in other modalities, functional hallucinations are usually given little diagnostic importance [2] and thus little is known about the treatment of these rare hallucinations. The treatment of positive symptoms of schizophrenia, including hallucinations, generally involves the use of antipsychotics. However, a number of patients do not respond even after several trials [3]. In such cases, other treatment methods need to be explored.

Electroconvulsive therapy (ECT) is one of the alternative strategies for its management. It induces a seizure lasting from 25 to 30 seconds with electric stimulus, given by electrodes attached to the scalp applied with or without anesthesia, namely, modified ECT (MECT) and direct ECT (DECT), respectively. It is generally given two to three times a week, usually for a total of eight to twelve treatment sessions in a series. In Nepal, DECT is still liberally used in many institutes mainly because it is relatively cheaper and less time consuming and requires few resources. To our knowledge four studies have been reported on ECT in Nepal. The studies have investigated clinical demographics of DECT and MECT receivers among psychiatry inpatients and compared improvement of patients who received ECT from those who did not. Schizophrenia has been the most common indication to receiving ECT in Nepal [4–7]. Here, we report the response of persistent functional hallucinations to ECT in a patient diagnosed with schizophrenia and discuss the role of ECT in this rare phenomenon.

## 2. Case Report

A 24-year-old girl from a low-middle class family studying medicine without any past and family history of psychiatric illnesses and well-adjusted premorbid personality presented to an out-patient department (OPD) five years back with complaints of hearing voices talking about her and commenting on her acts. She believed that people were talking about her whenever she saw them talking. Also, she heard male voices commenting on her or talk about her whenever there were other sounds like that of a spinning fan, a central processing unit (CPU) of a computer, or an aircraft. There was no history of substance use or mood disturbances. During the first year of the continuous illness, she had sought medical help from many centers for her problem and had used a number of antipsychotics. She had been on various antipsychotics with frequent changes due to their side effects rather than effectiveness. When the patient first visited our OPD she was on long-acting fluphenazine 25 mg IM every month, trifluoperazine 5 mg and trihexyphenidyl 2 mg twice daily, and amisulpiride up to 400 mg daily since at least one year, olanzapine 20 mg daily since about five months, and risperidone 3 mg daily. She had developed side effects like salivation and rigidity and weight gain. So, all the antipsychotics were tapered off except for fluphenazine and risperidone. However, she had become noncompliant due to her weight gain and stigma for the illness and was staying at home discontinuing her studies.

On examination, she was obese (BMI 28.8 kg/m^2^ with height of 150 cm and weight of 65 kgs). Routine laboratory investigations were unremarkable except for thyroid function test. Initial thyroid stimulating hormone (TSH) was 18.7 IU and the latest was 2.64 IU. Mental state examination revealed functional auditory hallucinations, audible thoughts, and delusions of reference. She was diagnosed to have paranoid schizophrenia with hypothyroidism and was treated with risperidone (titrated up to 6 mg/day) and long-acting fluphenazine 25 mg once a month due to compliance issues and thyroxine (titrated up to 75 mcg/day). With the medications, she improved significantly and was able to restart her studies, though she studied some other course. The brief psychiatric rating scale (BPRS) score, which initially was 43, was reduced to more than 70% in response to treatment. The only symptom persisting was the functional hallucination which was problematic to the extent of considering quitting all her medications believing this will never go away. Her father had work related to press at home. So, whenever she heard these machines roaring, she would hear male voices talking, abusing, and criticizing her. Also, the sounds made by the CPU of her computer brought the functional hallucinations causing a disturbance in her work. She did not hear these voices at any other time, nor did she have other symptoms. The patient had stopped taking risperidone on and off due to her obesity and was in distress. The patient and family were advised for ECT because of poor compliance and financial difficulties, as an adjunct to risperidone in the present dose (4 mg daily).

DECT was chosen by the patient and family as the method of ECT. The reason for using DECT was the financial restraints and the time management. DECT was cheaper (NPR 300) compared to MECT (NPR 1000), which required an arrangement with operation theatre and an anesthesiologist team. MECT also took more time than DECT, which hampered the work of patient's attendant. After the consent, DECT was given from a Medica Brief Pulse ECT machine (BPE 791), which provides a bidirectional brief square pulse with constant current (0.8 A). The charge was determined by titration method. The seizure was considered effective if the duration was at least 25 seconds. During the ECT, pulse width (1 ms), frequency (70 HZ), and stimulus train duration (1 s) were maintained. The seizure induced was tonic-clonic in type lasting for 35 (min) to 50 (max) seconds. The patient was cooperative for each ECT session without any apprehension towards the procedure. She complained of body ache after ECT but no other problems were observed or reported including memory deficit. An improvement of the functional hallucinations was noticed after the 3rd ECT session. With the plan of future maintenance ECT the electroconvulsive treatment was stopped at the 6th session. The patient improved significantly and remained stable on risperidone 4 mg/day for a few months but was then lost to follow-up. She came back after a year as her functional hallucinations started occurring occasionally. This time she had no problem with compliance nor was the functional hallucinations as bothersome. She agreed on continuing her medicine and to come for ECT and follow-up if it became problematic.

## 3. Discussion

Hallucinations form a predominant psychopathology in schizophrenia. It can occur in any of the sensory modalities. In the majority (70%) of the cases it is auditory, and in 50%, it is visual [8]. Functional hallucinations are less common. Nevertheless, they are problematic and disturbing to the patient needing relief from it. In our patient, auditory hallucinations occurred along with the sounds of CPU/fan, and in the absence of such stimuli, there were no hallucinations. The true hallucinations probably originated from abnormal activation of auditory cortex, and it has been suggested that in functional hallucinations there may be a misinterpretation of the neural encoding of natural-sound object and location characteristics that lead to the false perception that retains certain acoustic features of the original signal [9]. Usually functional hallucinations have little diagnostic importance [2]. However, this case illustrates the need to think about its significance in terms of diagnosis and management.

Schizophrenia with persistent functional hallucination is challenging to manage. Evidence-based treatments, such as changes in the medication [3] or cognitive-behavioral therapy [10], may not always be feasible. In the patient described here, compliance, side effect profile, and financial constraints reduced the conventional treatment options, requiring an alternative treatment method which was safe, affordable, and with some evidence of effectiveness. However, there is a dearth of literature on the management of functional hallucinations. A few case reports have suggested that carbamazepine and sodium valproate may be helpful [11]. Also, there are case reports suggesting the role of ECT as a treatment modality in atypical psychosis [12–14]. On the contrary, the recent National Institute for Clinical Excellence (NICE) guideline reports that “the current state of the evidence does not allow the general use of ECT in the management of schizophrenia to be recommended” [15]. Though ECT is a well-established psychiatric treatment method and had remarkable outcome during the 1930s, it is one of the stigmatized aspects of psychiatry and ECT is still the last choice of modality for treatment in schizophrenia. However, the literature is not consistent with the use of ECT in schizophrenia in developing and developed nations. A survey of the practice of ECT in Asia reports that schizophrenia is the most common diagnosis for which patients receive ECT [16]. The studies on ECT conducted in Nepal too show schizophrenia as the most common psychiatric condition for receiving ECT [4, 5, 7]. There are few studies assessing the effect of ECT in psychosis and hallucinations. Tharyan and Adams's systematic meta-analysis of double-blind randomized studies comparing ECT and antipsychotic medication to sham and medication revealed that ECT was better for the global improvement and initial period [17, 18]. The controversy and inconsistency regarding the use of ECT for persistent hallucinations in psychosis still exist. There is not any study demonstrating a specific relief of hallucinations in medication-resistant schizophrenia by ECT. Though ECT is considered as one of the augmentation strategies for the treatment of medication-resistant psychosis, the role of ECT in the core psychotic symptoms and hallucinations per se is not unclear [8].

Nevertheless, in our patient, the rare functional hallucinations decreased in severity with each session of ECT, highlighting the role and effect of ECT in resolving functional hallucinations. This case emphasizes ECT as an alternative treatment for functional hallucinations and possibly hallucinations in general. Although it cannot be recommended in all patients, this case suggests that ECT may be beneficial in selected cases, particularly as an add-on to antipsychotics. In low-income countries, ECT can be used as a more rapid treatment strategy for affective as well as a chronic psychotic disorder like schizophrenia. ECT is less expensive, safe, and rapid than antipsychotics in developing nations with minimal social service funds. Though antipsychotic drugs are still the first choice for schizophrenia treatment, there is no clear evidence to refute the use of ECT in the treatment of schizophrenia. More importantly, ECT worked tremendously well for this less prevalent but intriguing and troublesome psychopathology, functional hallucinations. So, why not ponder over the use of ECT as a treatment for both functional hallucinations and hallucinations in general.
